# Exploring Connections among Ecosystem Services Supply, Demand and Human Well-Being in a Mountain-Basin System, China

**DOI:** 10.3390/ijerph17155309

**Published:** 2020-07-23

**Authors:** Bojie Wang, Haiping Tang, Qin Zhang, Fengqi Cui

**Affiliations:** 1 Ministry of Education Key Laboratory of Ecology and Resource Use of the Mongolian Plateau, School of Ecology and Environment, Inner Mongolia University, Hohhot 010021, China; wangbj@imu.edu.cn; 2 Faculty of Geographical Science, Beijing Normal University, Beijing 100875, China; zhangqinbz@mail.bnu.edu.cn (Q.Z.); zhcuifengqi@163.com (F.C.)

**Keywords:** ecosystem services supply, ecosystem services demand, subjective well-being, mountain-basin area, local perception

## Abstract

Stakeholder perception and supply–demand relations are the main challenges and future directions for research on ecosystem services (ES). Based on spatial data and statistical data, we mapped eight key ES supply between 2005–2015 in the Huailai mountain-basin area. Using data from 507 survey questionnaires, we identified the ES demand and the public perceptions of the changes in ES. In addition, we also reveal the characteristics of the spatial distribution of ES demand, analyze the spatial supply–demand matching of ES, and explore the relationships between ES supply–demand and human well-being. From 2005 to 2015, a general upward trend was observed in the supply of four types of product provisioning services, which is different from the trend perceived by the stakeholders. An increasing trend was observed for carbon sequestration and forest recreation, which was in line with the perceptions of change. A spatial mismatch existed between the ES demand and supply, whereby the supply of carbon sequestration, soil conservation, habitat quality, and forest recreation services exceeded the demand in townships in the mountainous and hilly regions. On the other hand, townships located in the valley plains experienced a high imbalance between the demand and the supply. For the four types of product provisioning services, most towns and villages showed a balance in demand and supply. Linking ES supply–demand and human well-being showed that a threshold may exist in the supply–demand imbalance of regulating and supporting services before it begins to impact human well-being. Our study would enrich the theory and methodology research on relationships between ecosystem services and human well-being, and support knowledge to land allocation and management.

## 1. Introduction

Ecosystem services (ES) are the direct and indirect benefits that humans gain from ecosystem [1]. The assessments, trade-offs, and synergies of ES are the foundations of ES management and have attracted much attention [2,3,4]. As more in-depth studies become available, the links among ES supply, demand, and human well-being have become the new direction for research in this area [5,6]. Ecosystem service has emerged as a bridge between natural domain and social, human domain, which show both natural supply and human needs [7,8]. Therefore, the use of biophysics and geographical methodology to evaluate ES, combined with the use of sociocultural methodology to identify the stakeholders’ perception and preference of ES, are essential approaches to applying ES theory to ES management practice.

ES assessment cannot ignore the differences between the supply and demand of ES [9]. There is a growing interest in the studies of matching relationships between ES supply and demand in recent years [10,11,12]. Burkhard et al. initiated the matrix method in comparing ES supply–demand based on land cover [13]. Wang et al. developed a framework for integrating ES trade-offs/synergies to improve the match between ES supply and demand [9]. Xu et al. clarified the impacts of plantation on the changes between ES supply and demand based on emergy analysis [14]. Using questionnaire survey, existing studies quantified the mismatch between demand and supply of cultural services [15], and identified the ES supply and demand bundles by different stakeholders [16]. The participatory approach is based on the use of the stakeholders’ perceptions and preferences to analyze ES demand and supply, which can directly reflect the needs of the stakeholders and then can be used to promote effective system management [17]. Many studies highlight the importance of ecosystem services in sustainable landscape management and decision making [18]. Currently, more attention needs to be given to the spatial assessment and distinction of supply and demand matching, especially considering the subjective preference of stakeholders. 

Furthermore, it is important to understand the impact of such matching between ES supply and demand on human well-being [19]. Human well-being, as an endpoint for sustainability, was first linked with ecosystem services in Millennium Ecosystem Assessment (MA) [1,20]. Human well-being is based on benefits that are derived from ecosystem goods and services [1]. The supply of goods and services from an ecosystem should match the demands of society, which improve and sustain human well-being [13]. Previous studies have linked ES supply–demand and well-being and found that the mismatches in provisioning services had a strong impact on human well-being [5,21]. There have been several efforts to integrate ecosystem services and human well-being [8,22], yet the study of the relationships between ES supply–demand matching and human well-being is still lacking [21].

The mountain-basin system (MBS) features alternating mountains and basins in a multi-functional landscape [23]. The unique landform type of a mountain-basin system provides stronger spatial heterogeneity in its landscape features, where the ecosystem functions, socioeconomic compositions, and industries of each township have their own characteristics. Therefore, the MBS faces a deficit and mismatch between ES supply and demand across its diverse landscape. The Huailai MBS is close to China’s capital city of Beijing and is located upwind of Beijing. In the context of planning for the collaborative Development of Beijing, Tianjin and Hebei released by the Chinese government, Huailai County is assuming responsibility for the critical ecological support of Beijing [24]. 

The goal of this research is to inform local management of ES. We used township administration as a base unit to examine spatial and temporal changes in ES, as well as stakeholder perceptions of these changes. We quantified ES supply and demand, explored spatial relationships between supply and demand, and examined the links between supply–demand mismatches and human well-being.

## 2. Methods

### 2.1. Study Area

**Figure 1:**
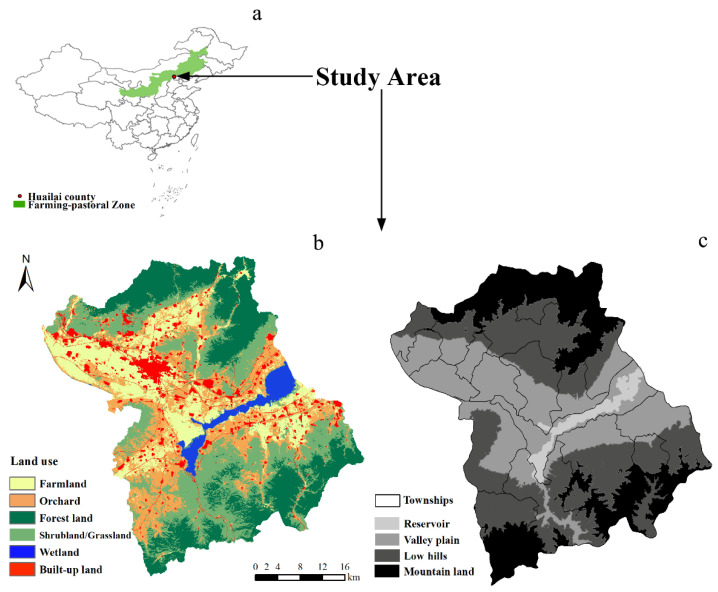
Location of (**a**) farming pastoral zone, (**b**) land use type, and (**c**) Huailai mountain-basin area.

The Huailai MBS is located in the south-east of the farming-pastoral area and covers an area of 1801.08 km^2^ (Figure 1a). Average annual precipitation at Huailai station was 391 mm, and average annual temperature was 9.2 °C. The main land use types include agricultural land (including orchard and farmland), forest land, shrubland/grassland, built-up land, and wetland, each accounting for approximately 39%, 24%, 28%, 6%, and 3% respectively (Figure 1b). The region has a complex topography and the elevations ranging from 394 to 1978 m above sea level, with the Guanting Reservoir in the middle. Tang and Zhang [25] have proposed a paradigm in the context of the natural and socioeconomic characteristics, which includes three layers and five functional regions: mountain land, low hills, and the intermountain basin (valley plain and reservoir; Figure 1c). The five functional regions included an ecological conservation region, grassland and raising livestock region, efficient economic region in valley plain, artificial protective region, and reservoir [25]. Almost 356,000 people live in the study area, distributed in 17 townships. Most local well-being depends on the provision of ecosystem services. 

### 2.2. Ecosystem Services Supply and Demand Assessment 

#### 2.2.1. Quantifying Ecosystem Services Supply

This study combines the use of field trips to the research location with information on policy background and previous studies. Taking into account aspects of ecology, production, and life, we selected eight key categories of ES as our research objects. We quantified ES supply between 2005–2015 based on spatial data and statistical data. The four provisioning services (including crop production, fruit production, vegetable production, and meat production) were quantified using statistical yearbooks at the level of townships. We used percentage of forest cover as a proxy of forest recreation based on land use data [26].

We modeled Net Primary Productivity (NPP) to quantify the supply of carbon sequestration based on the process-based Carnegie–Ames–Stanford Approach (CASA) model [27]. The formulas can be expressed as:

*NPP(x, t)* = *APAR(x, t)* × *ε(x, t)*, (1)

*APAR(x, t)* = *SOL(x, t)* × *FPAR(x, t)* × 0.5, (2)

*ε(x, t)* = *T_ε1_(x, t)* × *T_ε2_(x, t)* × *W_ε_(x, t)* × *ε_max_*, (3)

where *NPP(x,t)* represents the net primary production of at location *x* and time *t, APAR(x,t)* is the canopy-absorbed incident solar radiation over a time period (MJ·m^−2^), and *ε(x, t)* is the light utilization efficiency (gC·MJ^−1^). *FPAR(x,t)* is the fraction of photosynthetically active radiation absorbed by the vegetation canopy; *SOL(x,t)* is the total solar radiation (MJ·m^−2^); *T_ε1_(x,t)* and *T_ε2_(x,t)* refer to temperature stress coefficients; *W_ε_(x,t)* is the water stress coefficient; and *ε_max_* refers to the maximum light-use efficiency of vegetation under ideal conditions(g C·MJ^−1^).

We used the soil conservation amount to estimate the supply of soil retention based on Revised Universal Soil Loss Equation (RUSLE) [28], and the model can be expressed as:

*A_p_* = *R* × *K* × *LS*, (4)

*A_r_* = *R* × *K* × *LS* × *C* × *P*, (5)

*SC* = *A_p_* − *A_r_*, (6)

where *SC* represents the soil retention capacity (t·ha^−1^·year^−1^), *A_p_* is the potential soil erosion, *A_r_* is the real soil erosion, *R* is the rainfall erosivity factor (MJ·mm·ha^−1^·h^−1^·year^−1^), *K* is the soil erodibility factor (t·ha·h·ha^−1^·MJ^−1^·mm^−1^), *LS* refers to the topographic factor, *C* refers to the dimensionless cover management factor, and *P* is the conservation practice factor.

The results of models were validated by the results of previous studies [29,30,31]. The supply of habitat quality was calculated by Integrated Valuation of Ecosystem Services and Tradeoffs (InVEST) Habitat Quality model. The InVEST model combines information on land use and land cover (LULC) and threats to biodiversity to produce habitat quality maps. Detailed calculated parameters refer to Tallis et al. [32], and the basic formulas can be expressed as:

(7) $$Q_{xj}=H_{j}\left(1-\left(\frac{D_{xj}^{z}}{D_{xj}^{z}+k^{z}}\right)\right)$$

where $Q_{xj}$ is the habitat quality in grid cell *x* that is in LULC *j*; $H_{j}$ represents the habitat suitability of LULC type *j*; $D_{xj}$ the total threat level in grid cell *x* with LULC *j*; and *k* is scaling parameters (or constants).

#### 2.2.2. Ecosystem Services Demand Assessment 

The academic definition of ES demand can be mainly categorized as follows: (1) ES demand refers to the total usage or consumption of ES by humans within a certain time frame and locality, and (2) ES demand refers to the expression of human preference for specific ES [33,34]. Based on the above parameters, our research will account for the total consumption of ES, as well as the level of demand based on personal and social preference. Population density can reflect the total demand for ES, and it is used as the indicator for ES demand [35]. Therefore, this research used a survey questionnaire to identify the subjective preference for various ES in the different townships, followed by the use of population density to quantify the ES demand. 

This research uses the data collected from August 2016 from 42 typical villages; the samples are widely distributed in the various functional zones within the Huailai MBS. Using random sampling, we conducted face-to-face interviews and questionnaires, with a total of 507 effective survey questionnaires being collected. Compared with the Huailai County Statistical Yearbook, the interviewed samples can basically reflect the demographic characteristics of Huailai County. The respondents’ sociodemographic characteristics are shown in Table S1. In the process of the questionnaire survey, we obtained prior consent from the respondents, which was followed by explanations of the meanings of the various ES to promote better understanding of the questionnaire and attain more accurate results. The interviews lasted from 20 to 30 min. The questionnaire contained the following aspects: (1) respondents’ basic sociodemographic information; (2) the respondent’s perception of changes in the ES trend over the previous 10 years, and (3) social demand for various ES on a scale of 1–5, with 1 being the least and 5 being the most (see Supplementary Material 1). 

Urbanization has rapidly led to the large difference in the population density among townships; therefore, we minimized the extent of drastic fluctuation by using the logarithmic method in statistics [36]. The formula of ES demand was as followed:

*X_i_* = lg(*x_i1_*) × *x_i2_*, (8)

where *x_i1_* is the population density in township i, obtained by dividing the population by the size of the township; *x_i2_* is the average value of subjective ES preference of township i. 

With the township as the base unit, using the natural breaks classification in the computer program ArcGIS, we reclassified the ES demand in the various townships and assigned a value (1, 2, 3, 4, or 5) to each category to obtain the spatial distribution of the level of ES demand.

### 2.3. Human Well-Being Assessment

We selected five domains of human well-being (basic material for a good life, health, security, good social relations, and freedoms and choice) defined in the Millennium Ecosystem Assessment [1]. Three to five indicators were selected for each domain based on previous social surveys and interviews of the study area. Thus, 19 indicators were included in the questionnaires. Using random sampling, we conducted face-to-face interviews and questionnaires to ask the 19 questions using a five-point Likert scale. The weighted average approach was used for calculating the mean value of each domain of well-being. A higher score represented a higher level of human well-being. The value of each domain of well-being was standardized on a scale of 0–1. The original indicators and questionnaire were detailed in Wang et al. [8].

### 2.4. Data Processing and Analysis

With the township as the base unit, we calculated the average quantity of ES per unit area of the various townships for the period 2005 to 2015 to indicate the ES supply capacity in each township. Using the Natural Breaks classification within ArcGIS, we reclassified the ES supply in the various townships and assigned a value (1, 2, 3, 4, or 5) to each new category, thus obtaining the spatial distribution of the ES supply. Natural Breaks classes are based on natural groupings inherent in the data, and are formed of variance-minimization classification [37]. The Natural Breaks algorithm was detailed in Smith et al. [37]. This reclassification method was selected to better represent the spatial distribution of ES supply and demand. A higher value represented a higher level of ES supply and demand. Lastly, we subtracted the ES supply and demand layers to mapping the (mis)matches and relationships between ES supply and demand. 

Among the four broad types of ESs in MA, the contribution of provisioning services to human well-being was relatively straightforward [1,38]. Hence, we divided the eight ESs selected into two categories. The first category was provisioning services, including crop production, fruit production, vegetable production and meat production. The second category was regulating, supporting and cultural services including soil retention, carbon sequestration, habitat quality, and forest recreation. The ES supply and demand values were standardized on a scale of 0–1, and the average value of two categories of ESs in each township was calculated. We depicted the ES supply and demand of each township in a scatter plot using R software (R Core Team). We then classified ecosystem services into four types using the median of two categories of ES supply and demand, respectively. Lastly, based on the normalized value of well-being, we used star plots to visualize the well-being characteristics of the four types of ES supply and demand in R. Each petal of the star plot corresponds to the domain of well-being, and its size represents the value of well-being. 

## 3. Results

### 3.1. Perception of Changing Trends in Ecosystem Services

**Figure 2:**
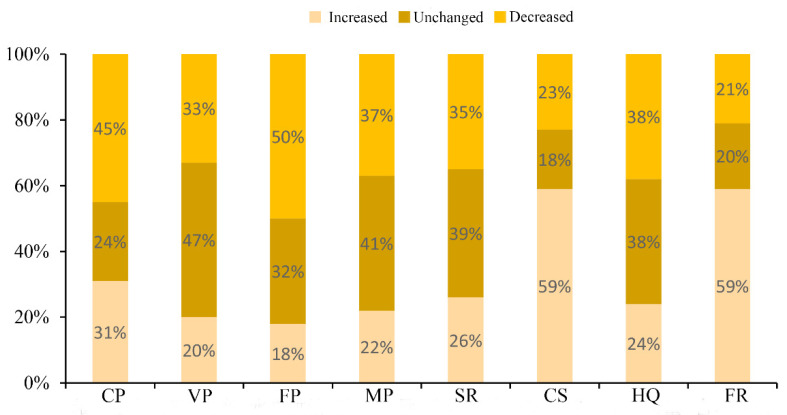
Huailai County stakeholders’ perception of changes in ecosystem services (ES) over the previous 10 years. CP: crop production; VP: vegetable production; FP: fruit production; MP: meat production; SR: soil retention; CS: carbon sequestration; HQ: habitat quality; FR: forest recreation.

The stakeholder’s perception of trends in ES changes is illustrated in Figure 2. Among the four provisioning services, 45% and 50% of the respondents perceived a decline has occurred in the crop and fruit production, respectively, over the previous decade. Additionally, many respondents, 47% and 41%, perceived that vegetable production and meat production, respectively, remained unchanged. Regarding the perception of soil conservation and habitat quality, no significant differences were observed for these items. In addition, the results also showed that 59% of the respondents perceive that there had been an increase in carbon sequestration and forest recreation over the past 10 years. 

### 3.2. The Spatial–Temporal Pattern of Ecosystem Services Supply

**Figure 3:**
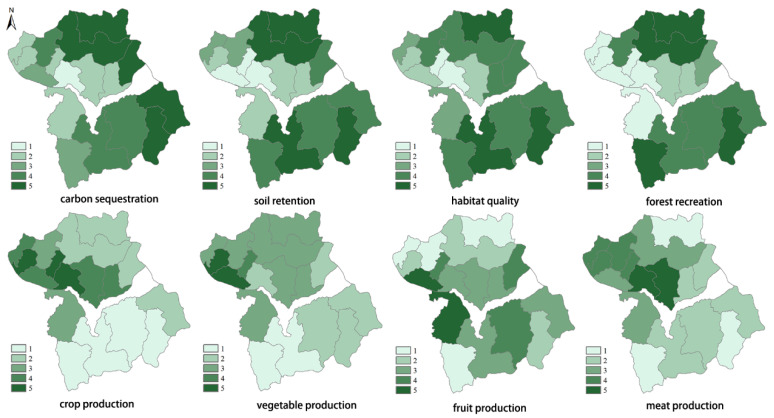
Spatial distribution of ecosystem services supply in Huailai county.

Carbon sequestration, soil conservation, habitat quality, and forest recreation were generally concentrated at the northern and southern mountainous and hilly regions, while a high supply of crop production was mainly distributed in the valley plains (Figure 3). The high value of vegetable production supply was mainly found in townships located upstream of the reservoir, with the supply in the northern mountainous and hilly area exceeding the supply in the southern area. Fruit provisioning was mainly located around the Guanting reservoir. The high values of meat provisioning were distributed in central of valley plain. 

An overall upward trend was observed in each ES item from 2005 to 2015. In 2005, 2010, and 2015, the annual average net primary productivity (NPP) was 367.39 gC/(m^2^·a), 373.48 gC/(m^2^·a), and 403.42 gC/(m^2^·a), respectively. The total NPP was 656,529.6 tC, 667,412.5 tC, and 720,915.6 tC, respectively, which shows an increase in carbon sequestration (Table S2). During the period 2005–2015, an increase in soil conservation was observed, with the first five years showing gradual growth in soil conservation per unit area, which increased from 116.92 t·hm^−2^a^−1^ to 129.93 t·hm^−2^a^−1^, and more significant growth in soil conservation between 2010 to 2015, rising from 129.93 t·hm^−2^a^−1^ to 164.57 t·hm^−2^a^−1^ and equivalent to an increment of 34.64 t·hm^−2^a^−1^. In the previous 10 years, the habitat quality improved. The ratio of the forestry area in Huailai County also increased. The proportion of area with habitat quality scoring between 0 and 0.2 and between 0.6 and 0.8 decreased 7.23% and 4.96%, respectively, while the proportion of area scoring between 0.2 and 0.4 and between 0.8 and 1 increased 7.98% and 4.2%, respectively (Table S3). The crop production also increased in 2005–2015: for the first five years, a larger fluctuation was observed in crop production, with an average growth of –12.35%, and for the last five years, the fluctuation in crop production decreased, with average growth of 2.49%. Greater fluctuation was observed in the vegetable production from 2005–2010, and the average growth was 5.33%, whereas between 2010 to 2015, the increase in vegetable production was more significant, with an average growth of 10.13%. The fruit production increased in 2005–2015 with average growth of 7.4%. The meat production increased rapidly from 1.4×10^4^ t in 2005 to 3.6×10^4^ t in 2015 (Figure S1).

### 3.3. Ecosystem Services Social Demand 

**Figure 4:**
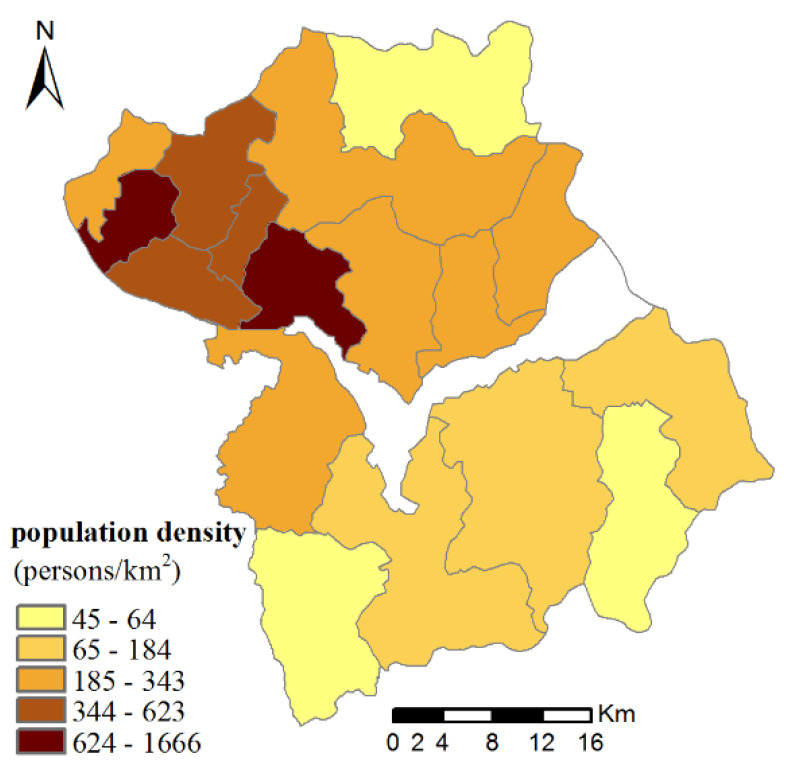
Spatial distribution of population density in Huailai county.

Due to the differences in landscape characteristics and socioeconomic development in every township, a significant spatial difference was observed in the population density of Huailai County. Townships located upstream of the reservoir have higher population densities. Shacheng Township was the socioeconomic and cultural center, where population density was far exceeding the other townships. The population density of the southern and northern mountainous regions was lower (Figure 4).

**Figure 5:**
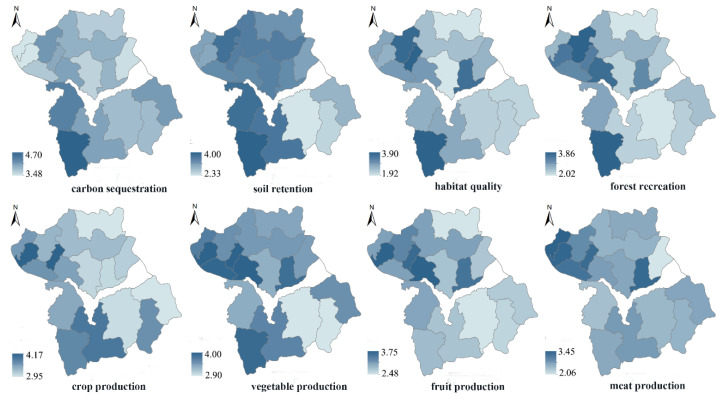
Spatial distribution of subjective preference based on the survey questionnaire.

The survey results show that spatial differences exist in subjective ES demand (Figure 5). The carbon sequestration demand in the various townships in Huailai county was high, with scores between 3.48 and 4.70, of which, the demands from the townships located in the south-western mountainous and hilly region were the highest. The demand for soil conservation service was higher among townships located in the south-western region. The demands for habitat quality and forest recreation were more dispersed, and Sunzhuangzi village in the south-western mountainous region had a high demand for both services. The townships located in the western river valley plain, as well as in the south-western mountainous region, had a higher demand for crop production and vegetable production services. The townships located in the flatland and hilly regions north of Guanting reservoir had a higher demand for fruit provisioning, whereas the townships located in north-western had a higher demand for meat production.

**Figure 6:**
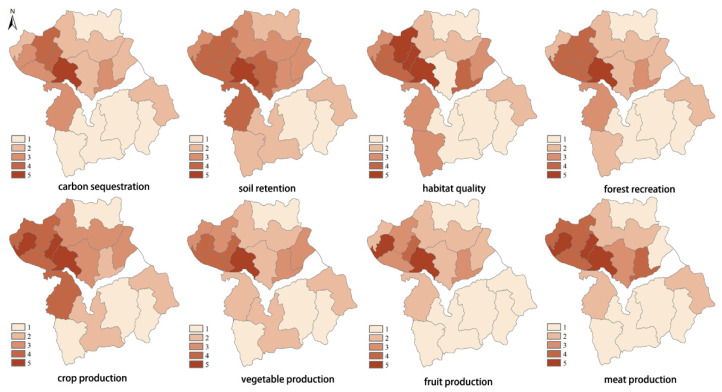
Spatial distribution of ecosystem services demand in Huailai County.

By combining the population density and the subjective demand preference for ES, we obtained the five levels of demand for ES (Figure 6). The results showed that higher demands exist for every ES item in the valley plain regions. The river valley plain region is characterized by flatland and by higher population density. The demand in the southern and northern mountainous regions is lower. By comparing the map for subjective demand and population density in each township, we saw that, although the demand for ES is determined by two factors—population density and subjective demand—the population density ultimately has a bigger impact on the ES demand, with the impact being greatest in Shacheng Township, which has the highest population density, and in Sunzhuangzi Village, which has the lowest population density. Therefore, in the management of ES, the focus should be placed on strengthening ES items with higher subjective demand and by improving ES provisioning in regions with high population concentration. 

### 3.4. Spatial Analysis of ES Supply–Demand Relations

**Figure 7:**
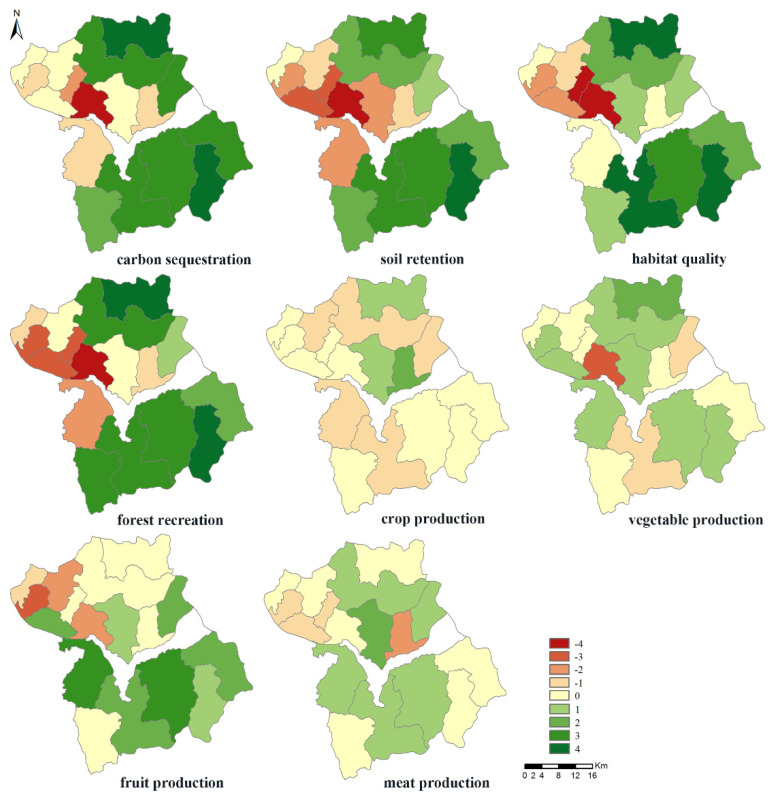
Spatial distribution of ES supply–demand relations in Huailai County.

From ES supply and demand regions in Huailai county, we obtained the spatial distribution of the ES supply–demand relations. The results showed that a spatial mismatch exists between the demand and supply of ES in Huailai County (Figure 7). For carbon sequestration, soil conservation, habitat quality, and forest recreation services, the townships where the supply exceeded the demand were located at the southern and northern mountainous and hilly regions. This is explained by the better growth of natural vegetation, high vegetation coverage, better habitat quality, and less soil erosion in the mountainous and hilly regions. On the other hand, the human activities in the river valley plains is more intensive, the population density is higher, the demand for ES is high, and the lands are mainly used for agricultural and construction purposes, creating a situation where the demand is higher than the supply, such as in Shacheng Township, which experiences the most severe supply–demand imbalance. In addition, apart from the north-western hilly regions’ fruit provisioning and Shacheng Township’s vegetable production, where the demand was still greater than the supply, most townships have relatively balanced relations between the demand and supply of the four product provisioning services. To summarize, a significant mismatch exists in the demand and supply of regulated services and cultural services in Huailai County, whereas the demand and supply of product provisioning services were relatively balanced. In the management of ES, to prevent the aggravation and expansion of imbalances, we need to consider the demand for regulating services and cultural services in densely populated townships in the river valley plain and manage, as early as possibly, the product provisioning services in the townships where the supply–demand shows an imbalance. 

### 3.5. The Relationships between ES Supply–Demand and Human Well-Being

**Figure 8:**
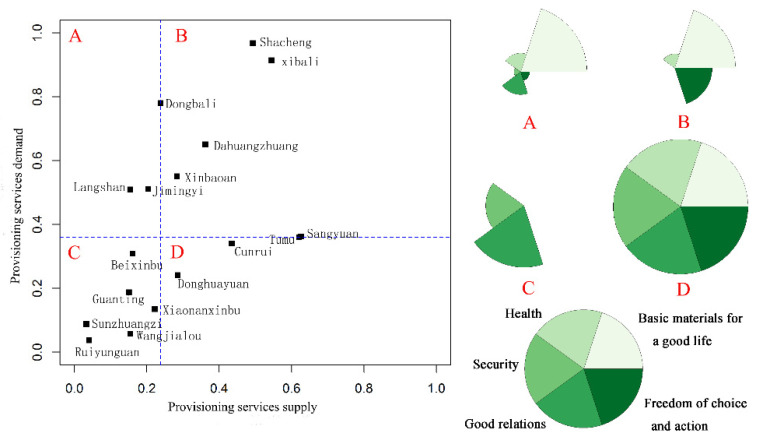
The well-being characteristics of four supply–demand types of provisioning services.

Most townships have relatively balanced relations between the demand and supply of the four provisioning services, which were classified into type B and C. The townships showed high supply and demand or low supply and demand. We analyzed the well-being characteristics of four supply–demand types of provisioning services. We found that the basic material for a good life was high in type A and B, and the freedom of choice and action was relatively high in type B. Type C was characterized by good social relations and high security, while other domains of well-being were lower. Type D had higher levels of all five domains of well-being (Figure 8).

**Figure 9:**
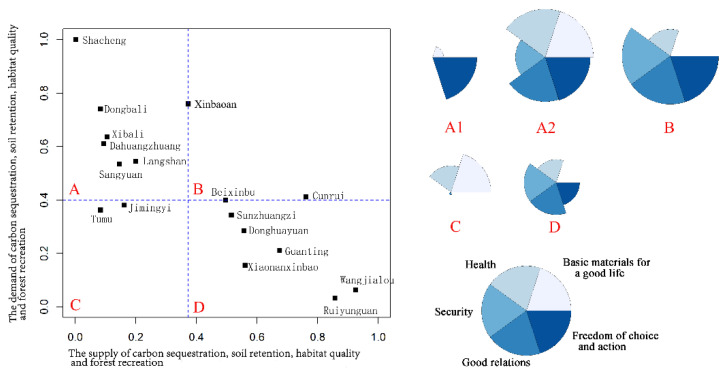
The well-being characteristics of four supply–demand types of carbon sequestration, soil retention, habitat quality, and forest recreation services.

For the second category of ES, including carbon sequestration, soil retention, habitat quality and forest recreation, most townships were classified into type A and D (Figure 9). The townships showed a mismatch between supply and demand of ES, which was characterized by high supply and low demand or high demand and low supply. We regarded Shacheng township as separate type A1 and other townships as type A2, due to the serious imbalance in Shacheng. The type A1 only had a high level of freedom of choice and action, while type A2 had higher levels of well-being domains except security. The type D was characterized by lower level of basic material for good life and general level of other domains.

## 4. Discussion

### 4.1. Stakeholder Participation in ES and Its Implications 

The participatory approach evaluates ES and social demand from another perspective. Via social feedback, this method allows an in-depth understanding of the scientific data acquired and provides more reliable scientific support for management and policy makers, which in turn form the channel for mutual support and communication among the public, science, and the government [39]. In addition, as ES support human well-being, an essential step for connecting ES and human well-being is an understanding of the preferences of the stakeholders and their perceptions of ES [40,41]. This research used a survey questionnaire to understand stakeholder perceptions of changes in ES. The results of the survey questionnaire and the estimated results of the biogeographic model and statistical analysis were taken as mutual references and integrated. The results from perceptions and models revealed that some similarities and differences appeared among ES indicators. A certain gap existed between stakeholder perceptions of changes in the four types of product provisioning services and our estimated results; this is perhaps due to the larger influence from personal factors on the perceptions statistics, where the respondents have a deeper impression on matters that have immediate impacts on their livelihoods and where drastic fluctuations in the product provisioning services over the years will influence the final perceptions [42]. The perception of changes in carbon sequestration and forest recreation was consistent with the estimated results, which generally show an upward trend. The respondents generally think that measures such as the restoration of farmlands into forest and the closure of mountainous area for reforestation are important for the improvement of the natural environment, and most respondents support these ecological conservation projects. These findings showed that subjective factors and respondent knowledge influenced the perceptions of ES, but the findings also show ES conditions can be observed from a different viewpoint; that is, critical input is provided to managers and policymakers. According to the results of our survey, we suggested that (1) local managers pay more attention to the drastic fluctuations in the product provisioning services to avoid livelihood risks and (2) local management could maintain the improvement of ES brought by ecological restoration projects. In addition, this study used the questionnaire survey method to determine the subjective demand for ES. Townships with fewer resources, such as Sunzhuangzi Village, tend to have a higher need for soil conservation, carbon sequestration, vegetable provisioning, and other services. The use of the questionnaire survey method to determine the subjective demand is advantageous for determining the demand for the regulating services and other services that are difficult to quantify; questionnaire survey data also supplement the footprint method, which is unable to provide individual calculation of the demand–supply relation for each item in ES, and consequently, the method used herein leads to more targeted management. This research serves as the initial idea and case study only, and more methods and case studies are required for the relevant topic. Future studies must also take into account the local environmental protection agencies and other relevant stakeholders.

### 4.2. The Balance of ES Supply–Demand and Human Well-Being 

Ecosystems provide ES through ecosystem processes and functions [43]. To satisfy their needs, humans have changed ecosystems and made choices that influence provisioning by ES, and the imbalances in the demand and supply for ES is a potential factor in the deterioration of ES [44]. Our findings showed that a spatial mismatch existed in the demand and supply for ES in our research area, in particular, the carbon sequestration, soil conservation, habitat quality, and forest recreation services in the river valley plains of the western region. The mismatches might derive from the unsatisfied demand and the existence of trade-offs between provisioning and regulating services [8,45]. The results suggest that managers should establish appropriate planning to regulate the mismatches between ESs supply and demand by reducing ES trade-offs. The critical target for ecosystem management is to increase the matching of ES supply–demand and improve well-being in multiple dimensions [9]. The supply–demand relations of ES have direct and indirect impacts on human well-being, and therefore, research on the demand–supply relations will enrich the studies on the relations between ES and human well-being [21]. Improvement in human well-being will be affected when human needs for ES cannot be satisfied. For instance, if the demand for a crop cannot be fulfilled, well-being will decline in terms of basic material needs; failure to meet the demand for forest recreation will influence higher level well-being factors. Overall, the level of well-being was high in valley plains but low in mountain land. The results showed that type D had higher levels of all five domains of well-being (Figure 8). The townships of type D were mostly distributed in valley plains, which were characterized by a high supply of provisioning services and a high level of well-being. We discovered that there was a severe imbalance in the supply and demand of regulating and supporting services in Shacheng Township, where the level of well-being was the lowest. However, the findings also showed that the level of well-being is not affected in townships where less imbalance exists between the demand and supply. The results might be due to the impact of regulating and supporting services on human well-being have a time lag and accumulating effect [26], and this indicates a threshold may exist in the supply–demand imbalance before it begins to impact human well-being. Local managers should take approaches to coordinate less mismatches between the ES supply–demand of townships in Type A2 (Figure 9) to avoid a huge decline in well-being. This study presents the relationships between the demand and supply for ES and human well-being from a new perspective. Future research should focus on the relations between the various factors that contribute to well-being and the supply–demand relations of the various ES, the contribution of ES flow to human well-being from the spatial perspective, and temporal analysis to determine whether a lag exists in the influence of the supply–demand relations of ES on well-being. 

### 4.3. Limitations

The findings presented should be considered in light of some limitations and uncertainties. We used questionnaire data to quantify ES demand and human well-being. The method was more appropriate at the local scale, but still subjective. The results were limited by the respondent knowledge and personal factors. Moreover, we only selected population density to estimate the total usage of ES, mostly due to lack of available data at the scale of our study. Finally, we did not account for the service flows, so future research should also consider how service flows and the benefits delivered to people are measured.

## 5. Conclusions

This study combines biophysics, geographical, and sociocultural methods to analyze the spatial and temporal changes of ES in the Huailai mountain-basin system and the relations between ES supply, demand, and human well-being, which may contribute to the improvement in the management of landscapes. The assessment results show that an overall upward trend existed in the various ES items from 2005–2015, of which the results for carbon sequestration and forest recreation were consistent with respondent perceptions, while some inconsistencies remain between the four product provisioning services and respondent perceived changes. A spatial mismatch exists between the ES demand and supply in the Huailai mountain-basin system. The demand of ES was higher than the supply in the river valley plains and especially in Shacheng Township, where the supply–demand imbalance was most serious, and the human well-being was also lower. A threshold may exist in the supply–demand imbalance of regulating and supporting services before it begins to impact human well-being.

## Supplementary Materials

The following are available online at https://www.mdpi.com/1660-4601/17/15/5309/s1, Table S1: Respondents characteristics, Table S2: Temporal changes of NPP, soil conservation, forest recreation, and habitat quality from 2005–2015 in Huailai County, Table S3: Habitat quality score in the year of 2000, 2010, and 2015 in Huailai County, Figure S1: Gross production of crop, vegetable, fruit, and meat from 2005–2015 in Huailai County.
