# Supplementary material for: Exploring Connections among Ecosystem Services Supply, Demand and Human Well-Being in a Mountain-Basin System, China
Source: Int J Environ Res Public Health. 2020 Jul 23;17(15):5309. doi: 10.3390/ijerph17155309 (PMC7432866; doi:10.3390/ijerph17155309)
Supplement: Supplementary file 1 [file ijerph-17-05309-s001.zip › Supplementary material-2.docx]

**Exploring connections among ecosystem services supply, demand and human well-being in a mountain-basin system, China**

Wang et al.

**Supplementary material 2**

**Table S1.** Respondents characteristics

|  |  | Total |
| --- | --- | --- |
| Sex | Male | 270 |
|  | Female | 237 |
| Age | <30 years | 84 |
|  | 30-49 years | 166 |
|  | 50-69 years | 233 |
|  | ≥70 years | 24 |
| Education | None | 84 |
|  | Primary | 170 |
|  | Secondary | 243 |
|  | University | 10 |
| Income (yuan/household/year) | <10000 | 155 |
|  | 10000-30000 | 211 |
|  | 30001-50000 | 90 |
|  | >50000 | 51 |

**Table S2.** Temporal changes of NPP, soil conservation, forest recreation and habitat quality from 2005-2015 in Huailai County

| Ecosystem services | 2005 | 2010 | 2015 |
| --- | --- | --- | --- |
| Annual average NPP (gC/m^2^) | 367.39 | 373.48 | 403.42 |
| Soil conservation (t·hm^-2^a^-1^) | 116.92 | 129.93 | 164.57 |
| Forest recreation (%) | 20.04 | 23.59 | 23.46 |
| Habitat quality | 0.59 | 0.61 | 0.61 |

**Table S3.** Habitat quality score in the year of 2000、2010 and 2015 in Huailai County

| Code | The interval values | 2005 | | 2010 | | 2015 | | Changes  in 2005-2015  （%） |
| --- | --- | --- | --- | --- | --- | --- | --- | --- |
|  |  | Area （ha） | Percentage（%） | Area （ha） | Percentage（%） | Area （ha） | Percentage（%） |  |
| 1 | 0-0.2 | 53925.21 | 30.23 | 41459.58 | 23.24 | 41022.63 | 22.99 | -7.23 |
| 2 | 0.2-0.4 | 25104.96 | 14.07 | 36125.55 | 20.25 | 39343.77 | 22.05 | 7.98 |
| 3 | 0.4-0.6 | 13.68 | 0.01 | 2.25 | 0.00 | 21.15 | 0.01 | 0.00 |
| 4 | 0.6-0.8 | 59275.71 | 33.23 | 55278.54 | 30.99 | 50434.65 | 28.27 | -4.96 |
| 5 | 0.8-1 | 40078.8 | 22.47 | 45525.06 | 25.52 | 47576.16 | 26.67 | 4.20 |


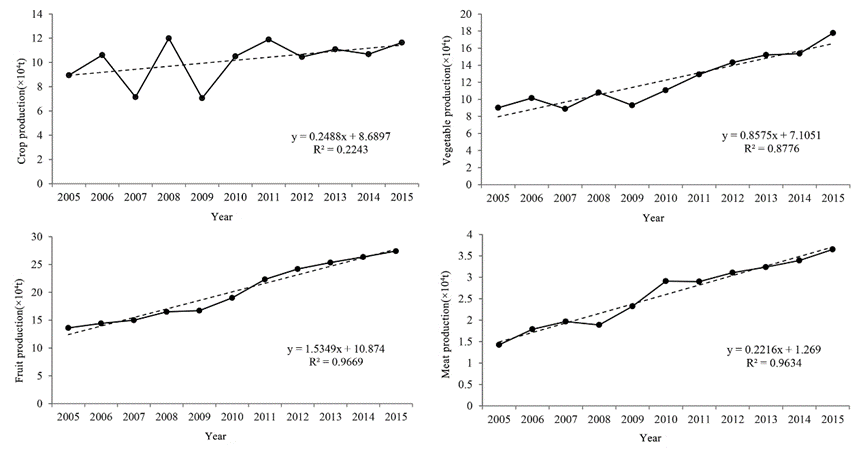


**Fig. S1.** Gross production of crop, vegetable, fruit and meat from 2005-2015 in Huailai County
