# Supplementary material for: Exploring Connections among Ecosystem Services Supply, Demand and Human Well-Being in a Mountain-Basin System, China
Source: Int J Environ Res Public Health. 2020 Jul 23;17(15):5309. doi: 10.3390/ijerph17155309 (PMC7432866; doi:10.3390/ijerph17155309)
Supplement: Supplementary file 1 [file ijerph-17-05309-s001.zip › Supplementary material-1.docx]

**Supplementary material 1. Questionnaire structure and content.**

1. **Respondents’ social-demographic information**

Township: _____________ Age: ______________ Gender: _______________

Family members: ___________

Which of the following intervals is your household annual income?

- < 10,000 yuan □ 10,000-29,999 yuan □ 30,000-50,000 yuan □ >50,000 yuan

What is your level of formal education?

- None □ Primary □ Junior high school

□ Senior high school □ University


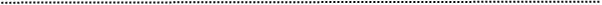


1. **Perception of changes in the ES trend**

For each service, how do you feel about the changes over the **previous 10 years**? Please select the corresponding option of changes in following table.

| Ecosystem services | Increased | Unchanged | Decreased |
| --- | --- | --- | --- |
| crop production |  |  |  |
| vegetable production |  |  |  |
| fruit production |  |  |  |
| meat production |  |  |  |
| soil retention |  |  |  |
| carbon sequestration |  |  |  |
| habitat quality |  |  |  |
| forest recreation |  |  |  |

1. **Information about the degree of demand for ecosystem services**

For each service, how do you feel about the demand for your everyday life? There are 5 degrees for each service, from 1 (lowest) to 5 (highest). Please rate the corresponding degree of demand in following table.

| Ecosystem services | 1 | 2 | 3 | 4 | 5 |
| --- | --- | --- | --- | --- | --- |
| crop production |  |  |  |  |  |
| vegetable production |  |  |  |  |  |
| fruit production |  |  |  |  |  |
| meat production |  |  |  |  |  |
| soil retention |  |  |  |  |  |
| carbon sequestration |  |  |  |  |  |
| habitat quality |  |  |  |  |  |
| forest recreation |  |  |  |  |  |
